# Sociodemographic predictors of flourishing among community older adults in China

**DOI:** 10.3389/fpsyg.2024.1435456

**Published:** 2025-01-20

**Authors:** Xin Yi, Xiaoyun Liu, Xiaoqun Liu, Gang Chen, Yujie Xie, Chenhao Lin, Hangqin Lv, Yingchun Li, Shuang Wu

**Affiliations:** ^1^School of Nursing, Chengdu University, Chengdu, China; ^2^Psychological Center, Chengdu University, Chengdu, China; ^3^The First Affiliated Hospital of Zhengzhou University, Zhengzhou, China; ^4^Department of Nursing, The Second People’s Hospital of Yibin, Yibin, China; ^5^School of Nursing, Chengdu Medical College, Chengdu, China

**Keywords:** flourishing, older adults, well-being, mental health, positive psychology

## Abstract

**Objective:**

With China becoming a rapidly aging society, the mental health of older adults has become an urgent concern. Past research on mental health assessment has primarily focused on psychopathological aspects such as depression or anxiety. In recent years, however, scholars in positive psychology have suggested that this may ignore the impact of individuals’ positive psychological qualities, such as well-being. “Flourishing” is a novel concept in the field of well-being. This adds to psychological well-being based on traditional social and subjective well-being. However, limited research has focused on the flourishing of older Chinese community-dwelling adults from the perspective of positive psychology. Therefore, this study explored the status and factors influencing flourishing among older Chinese community-dwelling adults.

**Methods:**

A total of 518 community-dwelling older adults living in Chengdu between January 2022 and March 2022 were included in this study through a cross-sectional survey. Participants completed the General Condition Questionnaire and the Flourishing Scale. The data were collected and recorded in a database using a two-person input format. Data analysis was conducted using SPSS Windows software version 26.0. Multivariate linear regression analysis was used to identify the factors influencing flourishing.

**Results:**

The mean score of the flourishing scale among Chinese community-dwelling older adults was 40.8 ± 8.8. Univariate analysis showed that economic condition, education level, spousal state, social participation, daily exercise, and chronic disease were significantly associated with flourishing among community-dwelling older adults. In contrast, gender, place of residence, and living state were not. Multivariate linear regression analysis showed that economic condition (*β* = 0.338, *p* < 0.001), education level (*β* = 0.300, *p* < 0.001), spousal state (β = −0.291, *p* = 0.008), social participation (β = −0.286, *p* = 0.013), daily exercise (β = −0.407, *p* < 0.001), and chronic disease (β = 0.313, *p* = 0.002) were significant influence factors of flourishing among community-dwelling older adults.

**Conclusion:**

This study is one of the first to explore the growth of community-dwelling older adults in China. The findings of this study make essential contributions to existing research on well-being. Interventions targeting older adults with poor economic conditions, lower educational levels, chronic diseases, limited social participation, and widowed or living alone are necessary.

## 1 Introduction

With the increasing aging population due to declining birth rates and rising life expectancies, maintaining and improving the well-being of older adults has become a critical strategic goal for global social and health policymakers, as emphasized by the World Health Organization (WHO) (Momtaz et al., 2011). In China, the government has addressed the mental health of older adults amid rising economic and social issues. However, in the assessment of mental health, most scholars in the past tended to evaluate mental health only from the perspective of psychopathology (e.g., depression and anxiety), which has gradually come into question over time (Keyes, 2002). As early as 1958, American psychologist Jahoda (1958) suggested that overreliance on negative indicators may lead to an overly narrow research perspective on mental health, thus neglecting the potential and self-repairing ability of individuals. Subsequently, researchers have proposed a dual-factor mental health (DFM) model.

DFM, first proposed by Greenspoon et al. (2001), utilizes psychopathological symptoms (depression, anxiety, etc.) as negative indicators and well-being as a positive psychological indicator of mental health. Keyes (2002) further categorized individuals into four groups: those with few psychological symptoms and high subjective well-being were characterized as flourishing, indicating complete mental health and resilience to future mental illness. Second, those with few psychological symptoms and low subjective well-being were categorized as languishing, indicating incomplete mental health and susceptibility to future psychological illnesses. Third, individuals with high psychological symptoms and high subjective well-being were described as struggling, indicating incomplete mental illness but with a robust self-repairing ability. Fourth, those with severe psychological symptoms and low subjective well-being were labeled as floundering, representing complete mental illness with psychopathological symptoms and poor psychosocial functioning (Huppert, 2009).

In recent years, well-being has become a research hotspot in positive psychology. According to a review of the relevant literature, existing research mainly conceptualizes well-being from eudaimonic and hedonic perspectives. From the eudaimonic perspective, well-being is fulfilling life’s purpose, meaning, and happiness. The hedonic perspective refers to high life satisfaction and positive affect (Hone et al., 2013). Studies have measured well-being only from eudaimonic or hedonic perspectives. However, researchers have found that well-being is best defined as a multidimensional concept that includes both eudaimonic and hedonic aspects (Ryan and Deci, 2001). Thus, the concept of flourishing has been proposed to reflect well-being comprehensively (Keyes, 2002).

Flourishing is a new field of well-being in positive psychology. According to American psychologist Keyes (2007), flourishing is a state of complete mental health, encompassing not only feeling good but also doing and living well. A flourishing person is characterized by enthusiasm, energy, positive emotions, and robust mental and social functioning (Seligman, 2011). This theory has garnered widespread attention since its proposal. Scholars from various countries have conducted relevant studies on flourishing. Studies in the United States, Russia, and Europe have focused primarily on students and the general population, resulting in varying levels of flourishing across countries. Studies have consistently demonstrated that flourishing significantly affects mental health, learning, and work efficiency (Bakker and Sanz-Vergel, 2013; Barber et al., 2010; Gokcen et al., 2012; Huppert and So, 2013; Westerhof and Taal, 2010). However, minimal research has been conducted on older adults’ flourishing.

In China, limited studies on flourishing have been conducted, mainly focusing on college and high school students, while there are no studies on community-dwelling older adults (Chen, 2015; Fang et al., 2018; Lai, 2014; Li, 2012). Therefore, this study explored the factors influencing flourishing among Chinese community-dwelling older adults. We hypothesized that the flourishing of Chinese community-dwelling older adults is in good condition, comparable to that in other Asian countries, but lower than that in European countries. Furthermore, we hypothesized that personal income, education level, spousal state, living state, social participation, daily exercise, and chronic diseases would be significant factors influencing the flourishing of older adults in Chinese community.

## 2 Methods

### 2.1 Study design and participants

The present study used a cross-sectional design and selected community-dwelling older adults from five cities in Sichuan Province over 3 months, from January 2022 to March 2022, using convenience sampling. Consent was obtained from all participants and their family members before completing the questionnaire, which took no more than 20 min. The inclusion criteria were: (1) aged 60 years or older and (2) Chinese citizenship. The exclusion criteria were as follows: (1) individuals experiencing delirium or suffering from mental disorders and (2) those who had recently participated in related surveys. The sample size was calculated for multivariate analysis using the following parameters: *n* = 1 + m + mψ^2^(1/*R*^2^−1). In the current study, *m* = 17, α = 0.05, ψ^2^ = 1.960, *R*^2^ = 0.298, yielding a calculated sample size *n* = 360. Allowing for a 10% inefficiency rate, the minimum sample size required was 396. The final sample size was 518. Further, this study was conducted in accordance with the Helsinki Declaration and the guidelines of the World Medical Association (WMA). This Ethics Committee of the Second People’s Hospital of Yibin approved this study (Protocol No. 2022-183-01).

### 2.2 Procedures

All participants completed the questionnaire at community health centers during assemblies focused on health education initiatives or free clinic programs. In each community, we employed two or three local staff members as data collectors after they received unified and centralized training. Face-to-face interviews were conducted with community data collectors. Considering older adults’ difficulties in completing the questionnaire independently and facilitating data collection, data collectors distributed e-questionnaires through Tablets to each participant. Participants with visual disturbances or illiteracy completed the questionnaire with the assistance of a data collector. The data collectors read the questions to the participants and completed the questionnaires based on their answers. Trained data collectors provided detailed instructions for the study before the participants completed the questionnaire. If participants had any doubts when filling in the questionnaires, the data collectors explained them until they clearly understood. Data collectors checked all questionnaires for completeness and accuracy. The questionnaire included general condition questions and a Flourishing Scale (FS).

### 2.3 Data analysis

All questionnaire data were entered into the database by two independent data collectors and analyzed using SPSS 26.0. Continuous data were reported as means and standard deviations, and categorical data were reported as frequencies and constituent ratios. In the univariate analysis, a *t*-test or variance test was used to identify the correlation between flourishing and all the variables. The multivariate linear regression analysis included variables with *p* < 0.05 in the univariate analysis to test factors related to flourishing among older adults.

### 2.4 Quality control

This study used an anonymous survey method to ensure anonymity and honest responses. Given that the respondents were older adults, we employed two to three community staff members as data collectors in each community. Extensive training sessions were provided to community data collectors to maintain the survey’s integrity and reduce bias. These sessions covered the purpose of the survey, the criteria for selecting respondents, and key considerations for conducting the survey. Additionally, community data collectors took a test after the training sessions. Only the participants who passed the test were allowed to complete the survey. This rigorous training regimen was implemented to ensure the accuracy and reliability of the data collection process.

However, owing to factors such as limited time and workforce, we used only random sampling to select cities, not communities. We endeavored to increase the sample size to maintain representativeness. Concerted efforts were made to control the risk of bias during the survey. For instance, the data collectors used unbiased language in explanations and collected questionnaires on the spot.

### 2.5 Measures

#### 2.5.1 General condition questionnaire

The researchers designed the general condition questionnaire, and data were collected on gender, place of residence, economic condition, education level, spousal state, living state, social participation, daily exercises (e.g., Tai Chi, dancing), and chronic disease.

#### 2.5.2 Flourishing scale

The FS was designed by Diener et al. (2010) and comprehensively describes well-being, including relationships, purpose, self-esteem, and optimism. The eight-item scale was measured using a seven-point Likert scale ranging from “strongly disagree” to “strongly agree.” Higher scores indicated higher levels of well-being. This study used the Chinese version of the FS introduced by Lai (2014). It has been confirmed to have high reliability and validity, with a Cronbach’s alpha of 0.948 and a test–retest reliability coefficient of 0.819. EFA revealed one factor that explained 75.03% of the total variation. CFA proved that all the goodness-of-fit indicators were acceptable (Zhang and Yang, 2018).

## 3 Results

### 3.1 Sample

Among 518 community-dwelling older adults, 241 (46.5%) were men and 277 (53.5%) were women. A total of 398 (76.8%) participants lived in urban areas, and 416 (80.3%) reported that their income satisfied their basic needs. Regarding educational level, 46 (8.9%) participants were illiterate, 90 (17.4%) had primary education, 236 (45.6%) had secondary education, and 146 (28.2%) had tertiary education. Of them, 423 (81.7%) were married. A total of 253 (48.8%) participants lived with their spouses, 220 (42.5%) lived with their children, 33 (6.4%) lived alone, and 12 (2.3%) lived in local nursing homes. Furthermore, only 88 (17%) older adults engaged in social participation, and 282 (54.4%) were involved in daily exercises such as Tai Chi and dancing. Additionally, 323 (62.4%) older adults reported suffering from a chronic disease (Table 1).

**Table 1:** Demographic characteristics and univariate analysis of community older adults.

Variables	Group	*n* (%)	FS ($\bar{X}\pm S$)	*t/F*	*p*-value
Sex	Men	241 (46.5%)	41.12 ± 8.78	0.810	0.418
	Women	277 (53.5%)	40.49 ± 8.87		
Place of residence	Rural	120 (23.2%)	40.56 ± 7.18	−0.372	0.711
	Urban	398 (76.8%)	40.86 ± 9.27		
Economic condition	Quite rich	32 (6.2%)	29.75 ± 11.02	26.797	<0.001
	Almost enough	416 (80.3%)	42.10 ± 7.78		
	Slightly poor	60 (11.6%)	37.18 ± 9.57		
	Quite poor	10 (1.9%)	43.00 ± 7.51		
Education level	Illiteracy	46 (8.8%)	32.93 ± 9.15	28.288	<0.001
	Primary School	90 (17.4%)	36.18 ± 9.06		
	Secondary	236 (45.6%)	42.01 ± 7.97		
	Tertiary	146 (28.2%)	44.13 ± 7.22		
Spousal state	Yes	423 (81.7%)	41.62 ± 8.29	4.081	<0.001
	No	95 (18.3%)	37.07 ± 10.13		
Living state	Solitude	33 (6.4%)	39.06 ± 8.36	0.709	0.547
	Living with children	220 (42.5%)	40.55 ± 8.90		
	Living with spouse only	253 (48.8%)	41.24 ± 8.77		
	Local nursing home	12 (2.3%)	40.25 ± 10.23		
Social participation	Yes	88 (17%)	45.26 ± 6.83	6.371	<0.001
	No	430 (83%)	39.87 ± 8.91		
Daily exercises (Tai Chi, Dancing, et al.)	Yes	282 (54.4%)	43.31 ± 7.67	7.356	<0.001
	No	236 (45.6%)	37.78 ± 9.18		
Chronic disease	Yes	323 (62.4%)	39.41 ± 9.19	−4.875	<0.001
	No	195 (37.6%)	43.07 ± 7.69		

### 3.2 State and influence factors of flourishing among community older adults

The mean score of flourishing among community-dwelling older adults was 40.8 ± 8.8. Univariate analysis of flourishing among community-dwelling older adults indicated that factors including economic condition, education level, spousal state, social participation, daily exercise, and chronic disease were significantly associated with flourishing. In contrast, gender, place of residence, and living status did not show a significant association with flourishing (Table 1). Furthermore, employing the essential factors from the univariate analysis as independent variables flourishing as the dependent variable, the multivariate linear regression analysis revealed that economic condition (*β* = 0.338, *p* < 0.001), education level (β = 0.300, *p* < 0.001), spousal state (β = −0.291, *p* = 0.008), social participation (β = −0.286, *p* = 0.013), daily exercises (β = −0.407, *p* < 0.001), and chronic disease (β = 0.313, *p* = 0.002) were significant influencing factors for flourishing among community older adults (Table 2). The factor values are listed in Table 3.

**Table 2:** Multivariate linear regression analysis of community older adults.

Factors	Unstandardized coefficients		Standardized coefficients beta	*t*	*p*-value	95%CI	
	β	SE					
Constant	34.947	3.201	–	10.918	<0.001	28.658	41.235
Economic condition	2.707	0.679	0.152	3.985	<0.001	1.372	4.042
Education level	2.401	0.273	0.354	8.804	<0.001	1.865	2.937
Spousal state	−2.325	0.871	−0.102	−2.668	0.008	−4.037	−0.613
Social participation	−2.289	0.916	−0.097	−2.499	0.013	−4.089	−0.489
Daily exercises (Tai Chi, Dancing, et al.)	−3.256	0.694	−0.184	−4.694	<0.001	−4.619	−1.894
Chronic disease	2.504	0.690	0.138	3.630	<0.001	1.149	3.860

**Table 3:** Factor values of the independent variables.

Variables	Value
Economic condition	0 = Quite rich, 1 = Almost enough, 2 = Slightly poor, 3 = Quite poor
Education level	0 = Illiteracy, 1 = Primary School, 2 = Secondary, 3 = Tertiary
Spousal state	0 = Yes, 1 = No
Social participation	0 = Yes, 1 = No
Daily exercises	0 = Yes, 1 = No
Chronic disease	0 = Yes, 1 = No

## 4 Discussion

### 4.1 State of flourishing

Few studies have explored the state and influencing factors of flourishing older adults worldwide, mainly in European and Asian countries. For example, the mean flourishing scores were reported as 39.2 in Finland and 46.7 in Croatia (Kainulainen, 2020; Kaliterna et al., 2002). The scores for Russian and New Zealand older adults were 40.9 and 45.2, respectively (Didino et al., 2019; Duncan et al., 2014). In Malaysia, the average flourishing score was reported to be 50.2, close to 52 in China’s neighboring country, Mongolia (Momtaz et al., 2016; Otgon et al., 2023). The current study revealed that the average flourishing score of community-dwelling older adults in China was 40.8.

Surprisingly, this result is discrepant with our previous hypothesis. Before the study, we hypothesized that the flourishing state among Chinese community older adults might be lower than that of European older adults but comparable to that of older adults in other Asian countries. However, the results of this study were significantly lower than those of the two Asian countries and slightly different from those of some European countries. One potential explanation for this could be cultural differences. Although all Asian countries share a similar cultural background, there are differences in cultural features, such as individualistic or collectivist cultures. It has been demonstrated that flourishing is related to cultural features; individualists score higher on flourishing and mental health than collectivists (Angel and Williams, 2013). Chinese culture, which is more collectivist, differs from the more individualistic culture of Mongolia (Wu et al., 2010). Another potential explanation is the demographic characteristics of the sample. Most community-dwelling older adults live in urban areas. They had good economic conditions and relatively high educational levels, contributing to both the eudaimonic and hedonic perspectives of well-being, resulting in a relatively good flourishing state.

### 4.2 Factors influencing flourishing

The multivariate linear regression analysis showed that flourishing among community-dwelling older adults was positively correlated with economic condition, education level, and chronic disease, and negatively correlated with spousal state, social participation, and daily exercise. Specifically, community-dwelling older adults with better economic conditions, higher education levels, a spouse, participation in social activities, engagement in daily exercise, and fewer chronic diseases showed higher levels of flourishing.

In this study, economic conditions refer to older adults’ self-awareness regarding the overall economic situation of their family. The results showed that community-dwelling older adults with better economic conditions had higher flourishing scores. Although few studies have explored the relationship between economic conditions and flourishing among older adults, relevant research reports have been found. For example, Otgon et al. (2023) reported that employed older adults have higher levels of flourishing than their unemployed counterparts, particularly those living in rural areas. The results are consistent with findings from other Asian countries, such as Malaysia and Eastern Thailand (Momtaz et al., 2016; Rojpaisarnkit, 2016). A possible reason for this is personal income from employment, as economic conditions in China fundamentally ensure individuals’ quality of life and significantly influence their sense of security and mental health.

This study found that the educational level of community-dwelling older adults was positively correlated with flourishing. In other words, community-dwelling older adults with higher education levels had higher flourishing scores, consistent with previous studies conducted in Asian countries, including Mongolia and Malaysia (Momtaz et al., 2016; Otgon et al., 2023). However, based on older American samples, Diener et al. (1995) indicated that education may not be closely related to well-being and flourishing. Given the limited number of studies reported thus far, we cannot confirm the relationship between education level and flourishing in older adults. Therefore, further studies with diverse samples across countries must verify the relationship between flourishing and education level.

Older community adults with spouses tended to have higher flourishing scores than those without spouses. For older adults, support from intimate family members is essential for survival and well-being. As an important component of the family social support system, it plays a vital role in coping with adversity (Didino et al., 2017). Additionally, older adults who engaged in social participation had higher flourishing scores compared to those who did not. Surveys on older Asian adults have reported similar results. Otgon et al. (2023) surveyed older Mongolian adults and reported that increased social activities and contact with friends contributed significantly to flourishing. Through structured interviews, van Schalkwyk and Wissing (2010) found that individuals with positive social relationships exhibited more self-confidence and positive coping, leading to flourishing. Older adults engaged in social participation can maintain social relationships with colleagues and friends, enhancing self-efficacy, self-worth, personal control, and well-being, thus contributing to a higher level of flourishing (Siegrist et al., 2004).

Community-dwelling older adults with chronic diseases reported lower flourishing scores. Chronic physical diseases often lead to negative emotions, such as anxiety, depression, and insomnia, which are closely related to mental health. Nabors et al. (2016) and Stough et al. (2015) explored the relationship between physical disorders and adolescent flourishing, finding that those with asthma and epilepsy had lower flourishing scores than their peers without physical disorders. Similarly, chronic pain in adolescents has been found to directly affect their physical, emotional, and social functions, thereby influencing their state of flourishing (Parsons et al., 2022).

### 4.3 Limitations

This study had several limitations. First, convenience sampling may have missed participants with poor health or mood. Second, the sample range was relatively limited, which may have affected generalizability. Future research should improve the sampling methods and expand the sample range to other provinces and cities. Additional potential influencing variables should also be considered in subsequent studies to facilitate a more comprehensive understanding of flourishing.

## Data Availability

The original contributions presented in the study are included in the article/supplementary material, further inquiries can be directed to the corresponding authors.
